# Iron overload and chelation modulates bisretinoid levels in the retina

**DOI:** 10.3389/fopht.2023.1305864

**Published:** 2023-12-22

**Authors:** Jin Zhao, Hye Jin Kim, Diego Montenegro, Josh L. Dunaief, Janet R. Sparrow

**Affiliations:** ^1^ Departments of Ophthalmology, Columbia University Medical Center, New York, NY, United States; ^2^ F. M. Kirby Center for Molecular Ophthalmology, Scheie Eye Institute, University of Pennsylvania, Philadelphia, PA, United States; ^3^ Pathology and Cell Biology, Columbia University Medical Center, New York, NY, United States

**Keywords:** iron, photoreceptor cells, retinal pigment epithelium, intravitreal injection, bisretinoid vitamin A, visual cycle

## Abstract

**Aim:**

Iron dysregulation in conjunction with other disease processes may exacerbate retinal degeneration. We employed models of iron overload and iron chelation to explore the interactions between iron-catalyzed oxidation and photoreactive bisretinoid lipofuscin.

**Methods:**

The mice were injected intravitreally with ferric ammonium citrate (FAC) or were treated using the iron chelator deferiprone (DFP) from birth to 2 months of age. Short-wavelength fundus autofluorescence (SW-AF) and spectral-domain optical coherence tomography (SD-OCT) scans were acquired. The bisretinoid levels were quantified using ultra performance liquid chromatography (UPLC) and *in vivo* through quantitative fundus autofluorescence (qAF). In histologic sections, the photoreceptor cell viability was assessed by measuring the thickness of the outer nuclear layer (ONL).

**Results:**

The levels of bisretinoids, all-*trans*-retinal dimers, and A2PE were significantly increased in the FAC-injected eyes of C57BL/6J mice. Seven days after FAC injection, hyperautofluorescent foci were visible in fundus autofluorescence (488 nm) images, and in SD-OCT scans, aberrant hyperreflectivity was present in the outer retina and ONL thinning was observed. In FAC-injected *Abca4^–/–^* mice with pronounced RPE bisretinoid lipofuscin accumulation, the hyperautofluorescent puncta were more abundant than in the wild-type mice, and the extent of ONL thinning was greater. Conversely, the intravitreal injection of FAC in *Mertk^–/–^* mice led to a more modest increase in A2PE after 2 days. In contrast to the effect of iron accumulation, chelation with DFP resulted in significantly increased levels of A2E and A2-GPE and qAF due to the reduced iron-catalyzed oxidation of bisretinoids. In *Mertk^–/–^* mice, the A2E level was significantly lower and the ONL area was smaller than in DFP-treated mice. DFP chelation did not impair the visual cycle in BALB/cJ mice.

**Conclusion:**

Iron accumulation was associated with progressive impairment in photoreceptor cells that was associated with the increased formation of a bisretinoid species known to form in photoreceptor outer segments as a precursor to A2E. Additionally, disease features such as the development of hyperautofluorescence puncta in fundus AF images, hyperreflectivity in the outer retina of SD-OCT scans, and ONL thinning were more pronounced when iron was delivered to *Abca4^–/–^* mice with a greater propensity for bisretinoid formation. Higher bisretinoid levels and enhanced qAF are indicative of lesser bisretinoid loss due to oxidation.

## 1 Introduction

Within the cellular and extracellular milieux, iron can exist in either ferrous (Fe^2+^) or ferric (Fe^3+^) states (1, 2). Iron present in the ferrous state functions in association with the hemeproteins hemoglobin and myoglobin, which reversibly bind oxygen. Ferrous iron is also associated with non-heme enzymes involved in oxidation–reduction reactions and the transfer of electrons (cytochromes and catalase). In cells, iron that is not being utilized is stored in ferritin; this is enabled by the oxidation of iron from its toxic Fe^2+^ state to its non-toxic Fe^3+^ state through the ferroxidase activity of the ferritin heavy chain, which allows it to be stored in the ferritin light chain (3). For the cellular export of iron by ferroportin and its subsequent binding to transferrin in plasma, Fe^2+^ is oxidized to Fe^3+^ by hephaestin and ceruloplasmin. Cells also maintain a cytosolic labile iron pool that is redox active, exists in the low micromolar range, and can bind to chelators (4, 5). Labile iron can also become toxic to cells due to the Fe^2+^-mediated generation of hydroxyl free radicals (this is known as the Fenton reaction). Under these conditions, iron chelation therapy has been employed using the drugs deferoxamine (DFO), deferiprone (DFP), and deferasirox to promote the excretion of chelated iron (6, 7).

The retina houses a family of bisretinoid fluorophores that constitute the lipofuscin of RPE (8). Bisretinoids form in photoreceptor cells due to the unchecked reactions of retinaldehyde with phosphatidylethanolamine (PE) (at a ratio of two to one) and are transferred secondarily to retinal pigment epithelial cells. These fluorophores include the pyridinium-containing molecules A2-glycerophosphoethanolamine (A2-GPE) (9) (A2, two vitamin A molecules), A2E and isomers of A2E (10–18), dimers of all-*trans*-retinal with a cyclohexadiene head group (all-*trans*-retinal dimers) (15, 19) and the associated protonated Schiff base conjugate (19), and A2-DHP-PE with an uncharged dihydropyridine ring (A2-dihydropyridine-phosphatidylethanolamine) (20). The bisretinoid A2PE is the immediate precursor of A2E, which forms in photoreceptor cells and undergoes phospholipase D-mediated enzymatic hydrolysis in RPE lysosomes (21–23).

When endogenous iron regulatory mechanisms are overwhelmed, such as in hereditary hemochromatosis or in individuals requiring repeated transfusion for the treatment of autosomal recessive beta-thalassemia (24), plasma iron levels exceed transferrin saturation, leading to the liberation of “free” iron. We have previously demonstrated that retinal degeneration due to iron overload can involve toxicity related to bisretinoid lipofuscin oxidation and degradation (25, 26). Bisretinoids are a unique target of hydroxyl radical-mediated oxidation because of the conjugated double bonds that form the side arms of these molecules (26). Thus, in the RPE cells of mice deficient in the ferroxidases ceruloplasmin and hephaestin, which convert Fe^2+^ to Fe^3+^ to enable Fe export, elevated iron levels were associated with decreased bisretinoid levels (26), a change reflecting oxidation-induced loss. Similarly, mice deficient in liver-specific hepcidin and exhibiting elevated iron levels in the neural retina and RPE due to reduced iron export exhibited a pronounced decrease in bisretinoid (measured as A2E) levels, accompanied by abnormal RPE morphology and photoreceptor cell degeneration (25). The latter is attributable to bisretinoid oxidation and degradation and the release of damaging dicarbonyls (26). Interestingly, recordings of fluorescence emission spectra from the RPE monolayer in LS-Hepc^–/–^ mice revealed that the peak fluorescence intensity was several fold greater in the mutant mice (25).

This study had two objectives. Ferric ammonium citrate (FAC) has previously been used to establish an iron-overload model that yields reproducible retinal damage (27). The first objective of this study was to demonstrate, using the intravitreal injection of FAC, that both RPE and photoreceptor cells are targeted by the iron-associated oxidative degradation of bisretinoids in the retina. The impairment of photoreceptor cells was monitored, alongside an increase in a specific species of bisretinoid, A2PE. The bisretinoid A2PE was detected only in photoreceptor cells (22, 28), and an increase in its formation could reflect the impaired handling of retinaldehyde. Iron chelation therapy is key to the management of iron overload, but the iron-binding efficiency of some iron chelators such as deferoxamine (DFO) is known to adversely impact this process due to the iron deficiency and associated effects on iron-dependent enzymes it brings about (29). RPEs in particular are known to suffer the adverse consequences of iron chelation caused by DFO (7).

## 2 Materials and methods

### 2.1 Mouse models

Eight-week-old adult wild-type black C57BL/6J mice, albino C57BL/6J*^c2j^* mice, and Balb/c mice were purchased from The Jackson Laboratory (Bar Harbor, ME, USA). Albino and agouti *Mertk* ^–/–^ mice were generated by mating B6;129-Mertktm1Grl/J (The Jackson Laboratory) with albino C57BL/6J^*c2j*^ mice (30). Albino and agouti *Abca4* null mutant mice (*Abca4* ^–/–^) were bred in the laboratory and housed under 12-h on–off cyclic lighting with in-cage illuminance of 30 lux–100 lux. All of the mice were homozygous for the Rpe65-Leu450 (*Abca4* ^–/–^ and Balb/c) or Rpe65-Met450 variant (C57BL/6J, C57BL/6J*^c2j^* and *Mertk* ^–/–^) (14). The choice of these mouse strains is explained in the Results section. The mutant mice did not carry the rd1, rdl2, rdl3, rdl6, rdl7, rdl8, and rdl10 mutations. All housing was designed, and all procedures were performed in accordance with the NIH’s *Guide for the Care and Use of Laboratory Animals* and the ARVO standards for the use of animals. The procedures were approved by the Animal Care and Use Committees at Columbia University and at the University of Pennsylvania.

### 2.2 Intravitreal injections

Fifty-two (52) mice (C57BL/6J, albino C57BL/6J^c2j^, albino *Abca4* ^–/–^ albino *Mertk* ^–/–^ mice) were injected intravitreally with iron in the form of FAC (MW 265; pharmagrade, Sigma-Aldrich, St Louis, MO, USA) (1 μL of 0.5 mM in 0.9% NaCl); injection sites were in the superior hemisphere near the ora serrata. Contralateral eyes (control) received saline only. After 2 days and 7 days the mice were studied.

### 2.3 Treatment with the iron chelator deferiprone

The BALB/cJ (*Rpe*65 Leu450) and agouti *Mertk* ^–/–^ (*Rpe*65 Met450) mice received the iron chelator DFP (Ferriprox) in drinking water (1 mg/mL). This began on postnatal day 1 and was initially conducted through the provision of the medicated water to the lactating mother; after weaning, the pups received DFP in their drinking water until they were 2 months old. The intake of DFP water was approximately 3 mL per day. The mice retinas were photobleached (white light) at 8,000 lux for 5 min and the retinoids were measured after 2 h.

### 2.4 Biomimetic synthesis

The A2E, A2GPE, A2-DHP-PE, atRALdi-PE, and A2PE species were synthesized and purified as previously described (9, 12, 19, 20). The purified bisretinoid species were redissolved and diluted in ethanol for UPLC analysis.

### 2.5 Quantitative high-performance liquid chromatographic and ultra performance liquid chromatographic analysis of bisretinoids

The whole mouse eyes (three or four eyes per sample as indicated) were homogenized and extracted in chloroform/methanol (1 : 1) and analyzed for bisretinoids (i.e., A2E, iso-A2E, A2-DHP-PE, and atRALdi-PE) using reversed-phase HPLC using an Alliance System (Waters Corp., Milford, MA, USA) and an Atlantis dC18 column, or a Waters Acquity UPLC-MS system and an Acuity BEH phenyl column (A2-GPE, all-*trans*-retinal dimer, and A2PE) (Waters, Milford, MA, USA), as previously described (31). The molar quantities per eye were calculated through comparison with synthesized standards. The levels of the pyridinium bisretinoid A2E and its *cis* isomer, iso-A2E, were measured separately and summed (A2E + iso-A2E).

### 2.6 Fundus imaging and histologic analysis

The fundus autofluorescence images (488 nm and 790 nm excitation) were obtained using a confocal scanning laser ophthalmoscope (Spectralis HRA; Heidelberg Engineering, Heidelberg, Germany). Short-wavelength fundus autofluorescence (SW-AF, 488 nm) was quantified as previously described (32). In anaesthetized mice (ketamine/xylazine) with dilated pupils (1% tropicamide, 2.5% phenylephrine hydrochloride) and corneal protection (GENTEAL^®^ gel; Alcon), high-resolution B-scan images of the retina were acquired using spectral-domain optical coherence tomography (SD-OCT; Bioptigen, Leica Microsystems, Buffalo Grove, IL, USA) as 1.8-mm radial and rectangular volume scans. The 5-µm paraffin-embedded H&E-stained sagittal sections that were most centrally located within the optic nerve head (ONH) were imaged digitally using a Leica AT2 whole-slide scanning system and analyzed as previously described (31).

### 2.7 Ultra performance liquid chromatographic analysis for retinoids

The mouse eyecups (one eye/sample) were homogenized and extracted in Dulbecco’s phosphate-buffered saline (PBS) containing 100 mM *O*-ethylhydroxylamine·HCl, neutralized to a pH of 6.5 using 4 N NaOH (33). The extraction process was performed on ice and under dim red light. After the addition of 1 mL of acetonitrile, all-*trans*-retinol acetate was added as an internal standard. To extract the retinoids from the homogenized tissues, hexane was added. After solubilization in hexane and centrifugation, the sample was dried under argon gas and redissolved in acetonitrile for UPLC quantification. The sample was injected into a reverse-phase column (CSH C18 column, Waters) for elution in a Waters Acquity UPLC system using gradients of water (A) and acetonitrile (B) containing 0.1% formic acid, as follows: 0 min to 5 min, 60% B; 5 min to 60 min, 60% to 70% B; 60 min to 70 min, 70% to 100% B; and 70 min to 90 min, 100% B (with a flow rate of 0.3 mL/min). The retinal (*O*-ethyl) oximes (11-*cis*-retinal and all-*trans*-retinal) were monitored at 360 nm, and the rest of the retinoids were monitored at 320 nm. The UV absorbance peaks were identified through comparison with external standards of synthesized retinoids.

### 2.8 Statistical analysis

The statistical analysis was carried out using GraphPad Prism, version 8 (GraphPad Software, Inc. La Jolla, CA, USA), and a *p*-value < 0.05 was considered significant.

### 2.9 Data availability

All the relevant data are provided in this paper.

## 3 Results

### 3.1 Bisretinoids in wild-type mice burdened with excess iron through intravitreal injection

We previously observed that in black wild-type C57BL/6J mice receiving iron in the form of FAC through intravitreal injection at 8 weeks of age, levels of atRALdi (detected through HPLC analysis using a C18 column 2 days after injection) were increased (27). To further investigate the iron-mediated damage in the retina both in photoreceptor cells and RPE, we delivered FAC intravitreally to 10-week-old C57BL/6J mice, harvested mice eyes 2 days after the delivery, and used a UPLC phenyl column to observe the changes in the other bisretinoid species. This phenyl column has short alkyl phenyl ligands covalently bound to the silica surface; as such, it typically lacks the hydrophobic retention that endows the stationary phase with higher levels of separation and resolution.

**Figure 1:**
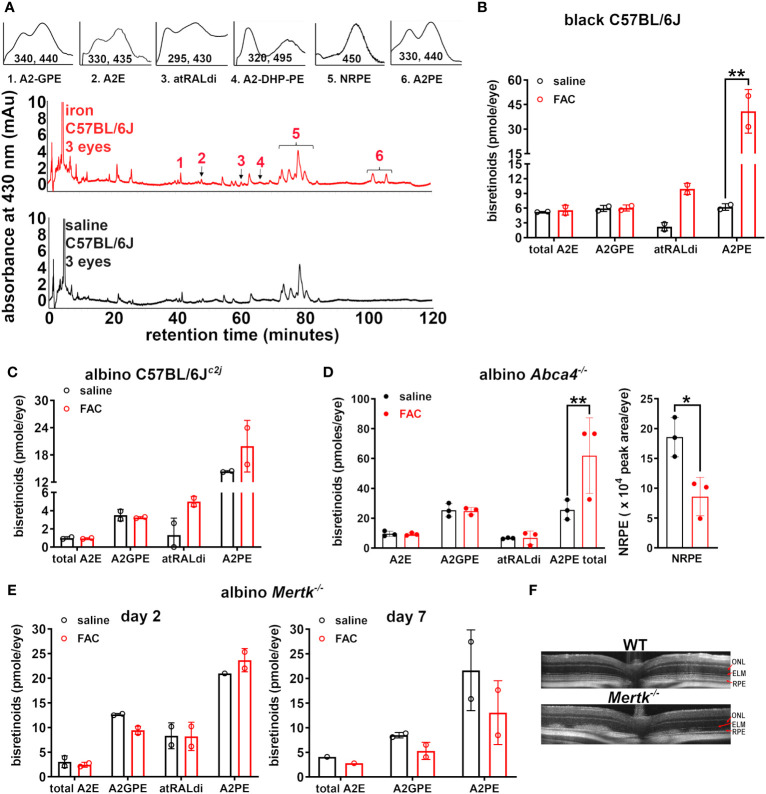
Analysis of bisretinoids in mice treated with ferric ammonium citrate (FAC) by intravitreal injection. The control mice were injected with saline. **(A)** Representative reverse-phase UPLC chromatograms presenting the detection of the bisretinoids A2E, A2GPE, A2-DHP-PE, atRALdi, and A2PE in the retinal extracts obtained from black C57BL/6J mice (aged 10 weeks) 2 days after the FAC injection. NRPE (*N*-retinylidene-phosphatidylethanolamine) is the Schiff base adduct formed by phosphatidylethanolamine and retinaldehyde. Insets above: UV-visible spectra of peaks corresponding to the compounds as indicated. The absorbance was monitored at 430 nm. **(B)** UPLC quantitation of bisretinoids in FAC- and saline-injected black C57BL/6J mice (aged 10 weeks, 2 days after the injection). The individual values are plotted with mean ± SD; a **p < 0.01, as determined by two-way ANOVA and Sidak’s multiple comparison test. **(C)** Albino C57BL/6J^c2j^ mice (aged 10 weeks, 2 days after injection). The individual values are plotted with mean ± SD. The values were not significantly different (*p* > 0.05). **(D)** The bisretinoid quantitation in FAC-injected albino *Abca4* ^–/–^ mice (aged 2 months) 2 days after injection. The graph on the right presents the quantitation of NRPE as a peak area per eye. Each value is based on the pooling of four eyes per sample. The individual values are plotted with mean ± SD; *p < 0.05 and **p *< *0.01, as determined by two-way ANOVA and Sidak’s multiple comparison test. **(E)** The bisretinoid quantitation in albino *Mertk* ^–/–^ mice 2 days and 7 days after FAC injection, aged 2 months. UPLC analysis. The mean ± SD (four eyes per value). **(F)** SD-OCT scans of uninjected wild-type (C57BL/6J^c2j^) and albino *Mertk* ^–/–^ mice.

The chromatographic identification of the bisretinoids A2-GPE, A2E, atRALdi, A2-DHP-PE, and A2PE in the UPLC profiles (**Figure 1A**) was enabled by the use of absorbance spectra and the adherence to retention times that corresponded to synthetic standards. The Schiff base *N*-retinylidene-PE (NRPE), which is formed by the reaction of retinaldehyde with PE was also detected. Notably, the levels of the bisretinoids atRALdi and A2PE were significantly increased, that is, 4.5- and 6.6-fold, respectively, in the iron-injected eyes of C57BL/6J mice relative to the saline-injected eyes of control mice (**p < 0.01, two-way ANOVA and Sidak’s multiple comparison test) (**Figure 1B**). However, the levels of A2E and A2GPE were not significantly different in saline-treated as compared with iron-treated mice.

Since albino mice are more prone to bisretinoid photodegradation (31), we also included these mice in our study. In albino C57BL/6J^c2j^ mice, a 1.4-fold increase in A2PE and a 3.8-fold increase in atRALdi were observed 2 days after the intravitreal injection of FAC (**Figure 1C**) although these differences, relative to saline injection, did not reach statistical significance (*p *> 0.05, two-way ANOVA, and Sidak’s multiple comparison test) (**Figure 1C**). We also noted that atRALdi and A2PE levels in albino C57BL/6J^c2j^ mice (**Figure 1C**) were lower than they were in the C57BL/6J mice (**Figure 1B**).

### 3.2 C57BL/6J: fundus imaging and light microscopic analysis

**Figure 2:**
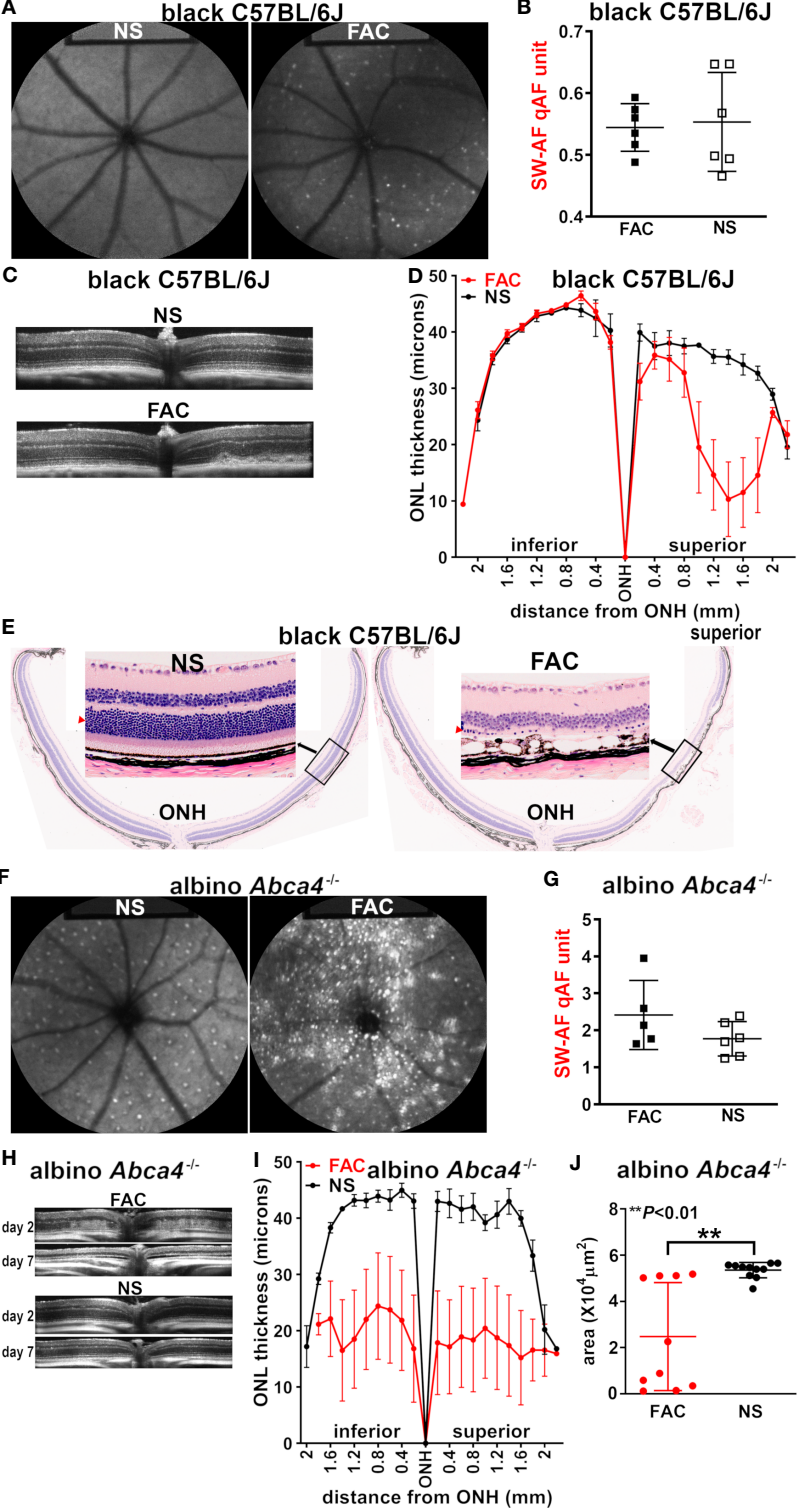
Multimodal imaging and analysis of the retina in mice injected intravitreally with ferric ammonium citrate (FAC) or normal saline (NS, control). The analysis was conducted 7 days after injection, unless otherwise indicated. **(A)** The representative *in vivo* fundus autofluorescence images acquired from FAC-injected and NS-injected C57BL/6J mice (aged 2–3 months). **(B)** The analysis of short-wavelength fundus autofluorescence by way of quantitative fundus autofluorescence (SW-AF qAF) in C57BL/6J mice (aged 2–3 months). The individual values are plotted with mean ± SD; *p* > 0.05, two tailed *t*-test. **(C)** The SD-OCT images acquired from FAC-injected and NS-injected C57BL/6J mice (aged 2 months). **(D)** The photoreceptor cell viability measured as outer nuclear layer (ONL) thicknesses in histologic sections of FAC- and NS-injected C57BL/6J mice and plotted at 0.2-mm intervals superior and inferior to the optic nerve head (ONH); the mean ± SEM of 10 eyes. **(E)** The representative H&E-stained sections of C57BL/6J mouse retinas (sagittal) injected with FAC or NS. The insets present magnified views of the indicated area (ONL, red arrowhead). **(F)** The *in vivo* short-wavelength fundus autofluorescence images acquired from NS- and FAC-injected albino *Abca4* ^–/–^ mice. **(G)** The qAF calculated using SW-AF images acquired from *Abca4* ^–/–^ mice. The individual values and mean ± SD are plotted; *p *> 0.05, unpaired two-tailed *t*-test. **(H)** The SD-OCT images from *Abca4* ^–/–^ mice 2 days and 7 days after FAC or NS injection. **(I)** The ONL thicknesses measured in *Abca4* ^–/–^ mice and plotted at 0.2-mm intervals superior and inferior to the optic nerve head (ONH). The mean ± SEM of 10 eyes per group. **(J)** The ONL area calculated using the ONL thicknesses (ONH to 1.6 mm in superior and inferior retina) measured in FAC- and NS-injected *Abca4* ^–/–^ mice. The mean ± SD of 10 eyes; the **p < 0.01 value was determined using an unpaired two-tailed *t*-test.

Bisretinoid lipofuscin is the source of SW-AF. Seven days after the intravitreal injection of FAC in C57BL/6J mice (aged 2–3 months), scattered hyperautofluorescent foci were detected using *en face* fundus autofluorescence (488 nm) imaging; these puncta were not present in the saline-injected mice, and in the FAC-injected mice, and were more prevalent in the non-central retina (**Figure 2A**). Nevertheless, these autofluorescent spots were not sufficient in number to cause an increase in SW-AF using the quantitative fundus autofluorescence approach (qAF) (**Figure 2B**) (32). Using SD-OCT, a non-invasive approach to assess the retina *in vivo*, B scans revealed the presence of aberrant disorganized hyperreflectivity in the outer retina, signifying damage to the outer retina (**Figure 2C**). The hyperreflective material was situated within the photoreceptor cell-attributable OCT bands and did not displace the photoreceptor-attributable bands, but rather replaced these outer retinal OCT bands. At the location of the hyperreflectivity, the ellipsoid zone and outer limiting membrane (ELM) were not distinguishable (as they were in the healthy eyes), and the thinned ONL was detectable anterior to the hyperreflectivity. The hyperreflectivity was more pronounced in the central retina.

Seven days post injection, the analysis of sagittally cut histologic sections revealed ONL thinning (**Figure 2D**), which was indicative of a reduction in photoreceptor cell viability in the superior retina, the location of the FAC injection. The ONL area (× 10^4^ μm^2^), calculated using the measurement interval of 0.2 mm multiplied by the sum of the ONL thicknesses in the superior hemiretina, was significantly reduced in the FAC-injected group (*p* < 0.05; mean ± S.D: saline-injected group, 7.12 ± 4.6; FAC-injected group, 3.7 ± 1.7). The loss of photoreceptor cells explains the absence of a difference in qAF intensity in FAC- vs. saline-injected mice (**Figure 2B**). Of the six FAC-injected eyes examined histologically, four exhibited ONL thinning in only the superior (dorsal) hemi-retina. In all FAC-injected eyes, the rows of nuclei in the ONL were reduced to one or two in the injected area (**Figure 2E**). In the superior hemisphere of the retina, the RPE monolayer presented with cell displacements, enlargements, and cell loss (**Figure 2E**), similar to our previous observations in LS-Hepc^–/–^ mice (25). These RPE disturbances may account for the areas of hypoautofluorescence in the fundus images (**Figure 2A**).

### 3.3 FAC injection in *Abca4* null mutant mice

To determine whether or not the effects of the FAC injection were related to the propensity for bisretinoid to form in retina, we injected 2- to 3-month-old albino *Abca4* ^–/–^ mice with FAC. *Abca4* ^–/–^ mice are characterized by the accelerated formation of bisretinoids in their photoreceptor cells, meaning that the accumulation in the RPE is also more copious (14). The A2PE levels were significantly higher in the FAC-injected albino *Abca4* ^–/–^ mice than in the saline-injected control mice (**Figure 1D**). For additional analysis of the A2PE data, we merged the A2PE data acquired from the C57BL/6J, C57BL/6J^c2j^, and albino *Abca4* ^–/–^ mice by normalizing the values for the FAC- and saline- injected mice with those of the saline-injected control mice. Accordingly, the final A2PE value of the FAC-injected eyes was 3.3-fold greater (*p* < 0.05, two-tailed *t*-test) than that of the saline-injected eyes (the mean ± SD was 3.3 ± 2.50 and 1.0 ± 0.15 for the FAC-injected group and saline-injected group, respectively).

We found that 7 days after iron injection, the prevalence of hyperautofluorescent puncta in the SW-AF images was substantially increased in the FAC-injected *Abca4* ^–/–^ mice (**Figure 2F**) relative to both the FAC-injected wild-type mice (**Figure 2A**) and the saline-injected *Abca4* ^–/–^ mice (**Figure 2F**). Nevertheless, given that the fundus presented with both hyper- and hypoautofluorescence foci, a statistically significant increase in the overall qAF relative to the control saline-injected *Abca4* ^–/–^ mice was not observed (**Figure 2G**). The OCT B scans revealed diffuse hyperreflectivity in outer retina 2 days post injection, with, in some cases, the almost-complete absence of the ONL 7 days after FAC treatment (**Figure 2H**). The measurements of ONL thickness at 0.2-mm intervals using histologic sections (7 days after injection), were plotted as a function of distance from the ONH inferiorly and superiorly (**Figure 2I**). The FAC-injected *Abca4* ^–/–^ mice exhibited pronounced but highly variable ONL thinning (**Figures 2I, J**).

### 3.4 Effects of intravitreally injected iron on subretinal bisretinoids in albino *Mertk^-/-^* mice

Until now we have largely focused on the interactions between bisretinoids that are amassed intracellularly in the RPE. We observed defective RPE-mediated phagocytosis of the outer segments, which led to the accumulation of bisretinoids in the outer segments of photoreceptor cells and the deposition of bisretinoids within the subretinal space in mice without the *Mertk* mutation (30). In these mutant mice, the intravitreal injection of FAC led to a modest increase in the A2PE level 2 days after injection, although the difference in the A2PE level observed between the FAC- and saline-injected mice was not significant (**Figure 1E**). When examined 7 days after the FAC injection, the A2PE level was considerably diminished (**Figure 1E**). In SD-OCT B scans, outer retinal degeneration was indicated by aberrant disorganized hyperreflectivity; the ellipsoid band and the external limiting membrane could not be distinguished (**Figure 1F**).

### 3.5 Iron chelation with DFP

As opposed to iron overload, the effects on the retina due to chelation-dependent iron-depletion are also of concern (7, 25). Among the iron-dependent enzymes of the retina, *Rpe*65 has an iron-binding domain and the catalytic activity of *Rpe*65 is dependent on the availability of iron (34). *Rpe*65 is the isomerase enzyme that converts all-*trans*-retinal ester to 11-*cis* retinol and is thus an essential enzyme providing 11-*cis* chromophore to photoreceptor cells (35). Thus, another interest of ours is whether or not iron chelation impacts levels of 11-*cis*-retinal.

**Figure 3:**
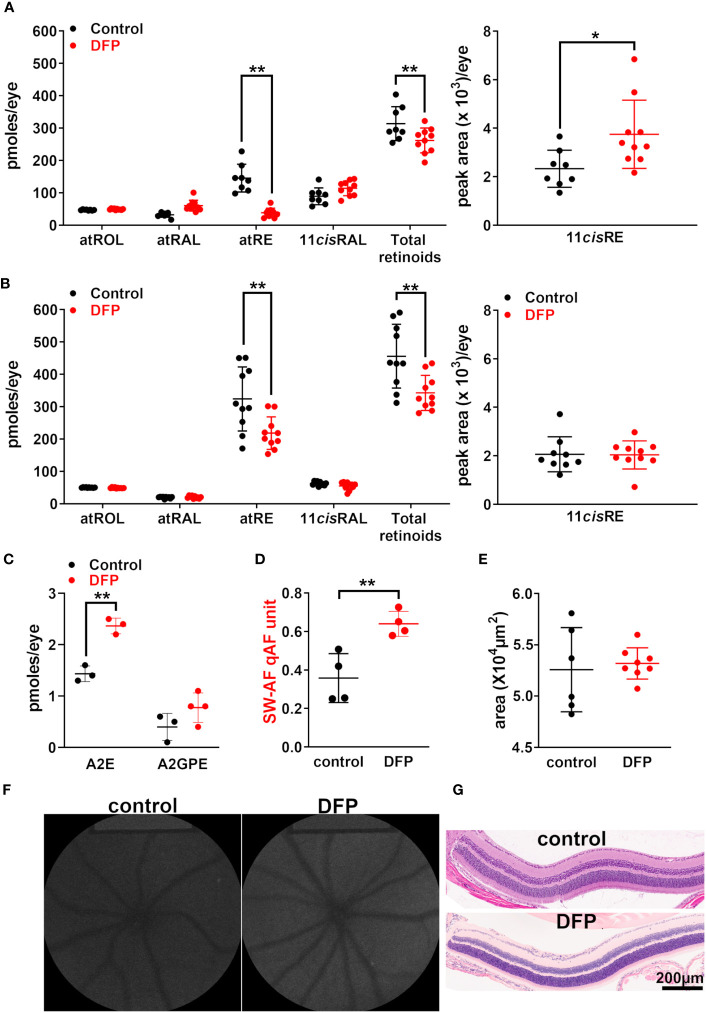
BALB/cJ mice treated with the iron chelator deferiprone (DFP) from birth. The control mice received no treatment. **(A)** The UPLC quantitation of the retinoids all-*trans*-retinol (atROL), all-*trans*-retinyl palmitate (atRE), all-*trans*-retinal (atRAL), 11-*cis*-retinal (11cisRAL) (pmoles/eye), and 11-*cis*-retinyl palmitate (11cisRE) (peak area x 10^3^/eye) 2 h after photobleaching of BALB/cJ mice (aged 2 months). The individual values are plotted with mean ± SD of eight to 10 eyes; *p < 0.05, **p < 0.01, two-way ANOVA and Sidak’s multiple comparison test. **(B)** The UPLC quantitation of retinoids in DFP-treated dark-adapted BALB/cJ mice (aged 2 months). The mean ± SD of 8–10 eyes; **p <0.01, two-way ANOVA and Sidak’s multiple comparison test. **(C)** The HPLC (A2E) and UPLC (A2-GPE) quantitation in the eyes of mice treated with DFP from birth until they were 2 months old. The individual values are plotted with mean ± SD; **p < 0.01, one-way ANOVA and Tukey’s multiple comparison test. **(D)** The intensities of short-wavelength fundus autofluorescence (quantitative fundus autofluorescence, qAF). Each value is the mean of one pair of eyes (four mice) with mean ± SD; **p < 0.01, unpaired two-tailed *t*-test. **(E)** The area of the outer nuclear layer determined in BALB/cJ mice treated with DFP and in untreated mice. The area was calculated using the ONL thicknesses 1.6 mm superior and inferior to the optic nerve head. The mean ± SD of six to eight eyes; *p* > 0.05, unpaired two-tailed *t*-test. **(F)** The representative *in vivo* short-wavelength fundus autofluorescence (488 nm excitation) images acquired from untreated and DFP-treated BALB/cJ mice (aged 2 months). **(G)** Photomicrographs of the inferior retina acquired from DFP-treated and control mice, aged 2 months.

Rodents do not have sight at birth; the generation of rods that constitute 95% of the photoreceptors in mouse retina continues until postnatal (PN) day 7 and the a-wave of the electroretinogram cannot be recorded until PN day 10 (36). To test for effects on *Rpe*65, we utilized BALB/cJ mice, as they express wild-type *Rpe*65 with a leucine residue at position 450. Thus, to reduce the iron availability during photoreceptor cell differentiation, we treated postnatal BALB/cJ mice by adding the iron chelator DFP (1 mg/mL; since the mice were born) to the mothers’ drinking water; this was followed by the mice directly ingesting DFP through their drinking water until they were 2 months old. We have previously shown that mice under the foregoing DFP-treatment protocol exhibit reduced iron in the retina, as measured by bathophenanthroline-based spectrophotometry (37) and by quantifying transferrin receptor mRNA, a measure reflecting the intracellular Fe concentration (26). In the DFP-treated mice under dark-adapted conditions (24 h), the all-*trans*-retinyl ester levels were reduced, and those of the 11-*cis*-retinyl ester were increased, whereas those of the other retinoids (i.e., 11-*cis*-retinal, all-*trans*-retinol, and all-*trans*-retinal) were not affected by early-stage iron chelation (**Figure 3A**). These changes in the retinyl ester content suggested that DFP exerted modest effects on the visual cycle. We also tested *Rpe*65 kinetics after DFP-chelation by measuring the recovery of 11-*cis*-retinal levels 2 h after photobleaching (90%). Once again, the levels of 11-*cis*-retinal were different between the treated and untreated mice, although the all-*trans*-retinyl ester levels were reduced after the 2-h interval (*p* < 0.001, two-way ANOVA and Sidak’s multiple comparison test) (**Figure 3B**). The bisretinoids form as a byproduct of the visual cycle and can serve as a longer-term indicator of visual cycle functioning. We observed that DFP treatment during the postnatal period and until the mice were 2 months old was associated with significantly increased A2E levels (**Figure 3C**) due to reduced iron-catalyzed oxidation of bisretinoids (25, 26). SW-AF, measured as qAF, also correspondingly increased (**Figures 3D, F**). No differences were observed in photoreceptor cell viability measured as the ONL area (**Figures 3E, G**).

### 3.6 DFP treatment of *Mertk* ^–/–^ mice

**Figure 4:**
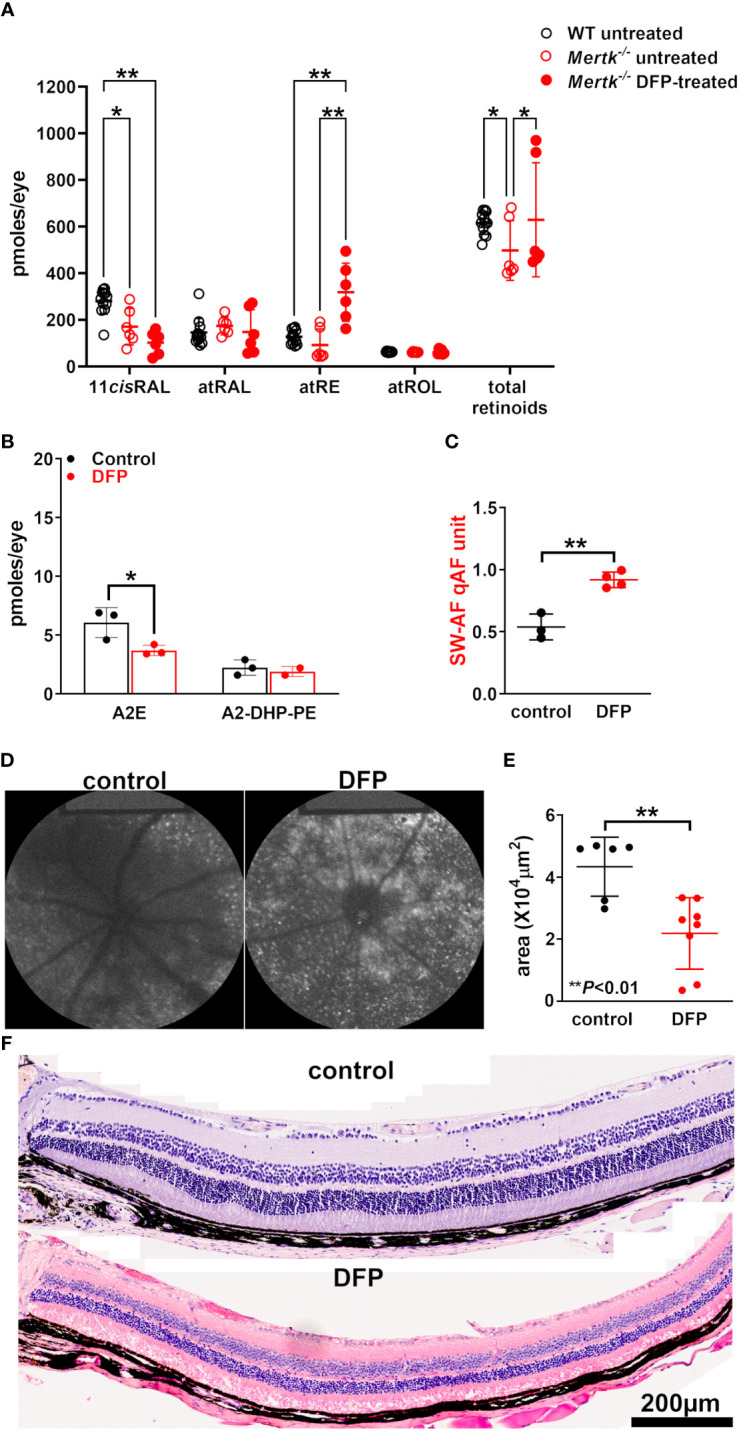
Agouti *Mertk* ^–/–^ mice treated with the iron chelator DFP since birth. **(A)** The retinoid levels in the light-adapted agouti wild-type mice, untreated *Mertk* ^–/–^ mice, and *Mertk* ^–/–^ mice treated with DFP from birth until they were 8 weeks old. The UPLC analysis: all-*trans*-retinol (atROL), all-*trans*-retinyl palmitate (atRE), all-*trans*-retinal (atRAL), 11-*cis*-retinal (11cisRAL). *p < 0.05, *p < 0.01, two-way ANOVA and Sidak’s multiple comparison test. **(B)** The bisretinoid quantification (HPLC analysis) of mice aged 2 months, 4–6 eyes per sample, and three samples; *p < 0.05, one-way ANOVA and Sidak’s multiple comparison test. **(C)** the SW-AF intensities calculated as quantitative fundus autofluorescence (qAF). The mean ± SD based on four mice; **p < 0.01, unpaired two-tailed *t*-test. **(D)** The representative *in vivo* short-wavelength fundus autofluorescence (488 nm excitation) images acquired from control and DFP-treated *Mertk* ^–/–^ mice (aged 2 months). **(E)** The area of the outer nuclear layer in agouti *Mertk* ^–/–^ mice calculated from thicknesses 1.6 mm superior and inferior to the optic nerve head. The mean ± SD of 6–8 eyes; **p < 0.01, unpaired two-tailed *t*-test. **(F)** The photomicrographs of the control and DFP-treated inferior retina acquired from *MertK^-/-^* mice aged 2 months.

The agouti *Mertk* ^–/–^ mice (untreated) at 8 weeks of age (i.e., 2 months) exhibit an appreciable subretinal increase in bisretinoids levels relative to wild-type mice, in addition to a reduction of approximately 20% reduction in the ONL area (30). Compared with those in wild-type mice, the 11-*cis*-retinal levels in our cohort of 2-month-old *Mertk* ^–/–^ mice (untreated) were significantly reduced under light-adapted conditions (*p* < 0.05, two-way ANOVA and Tukey’s multiple comparison test) (**Figure 4A**); however, we observed no difference in the levels of the other retinoids (*p* > 0.05, two-way ANOVA and Tukey’s multiple comparison test). In the *Mertk* ^–/–^ mice treated with DFP since birth, we observed a further significant reduction in 11-*cis*-retinal levels (by age 2 months) together with an increase in all-*trans*-retinyl ester levels (*p* < 0.001, two-way ANOVA and Tukey’s multiple comparison test) (**Figure 3F**). No differences were observed in the all-*trans*-retinol and all-*trans*-retinaldehyde levels (*p* > 0.05, two-way ANOVA and Tukey’s multiple comparison test). The treatment of the agouti *Mertk* ^–/–^ mice with DFP from birth to age 2 months yielded a 39% decrease in A2E levels (**Figure 4B**). Nevertheless, the qAF was increased (**Figures 4C, D**). In these DFP-treated agouti *Mertk* ^–/–^ mice, the ONL area was reduced (*p* < 0.01, unpaired two-tailed *t*-test) (**Figures 4E, F**); this decrease is suggestive of exacerbated photoreceptor cell death and may account for some of the loss of A2E (**Figure 4B**).

## 4 Discussion

It was demonstrated several years ago that lipid oxidation is not involved in the production of RPE lipofuscin formation (38, 39). For example, RPE lipofuscin fluorophores exhibit peak emissions at significantly longer wavelengths than the blue-emitting fluorescent products (stimulated using UV light) of lipid autooxidation (40). Nevertheless, lipid peroxidation is a lethal process linked to iron-induced cellular injury. In addition to the damage mediated by lipid peroxidation, the retina is burdened by the production of oxidant-generating bisretinoids that form in photoreceptor cells as a byproduct of the visual cycle (25, 26, 33). Cellular damage ensues from bisretinoid oxidation due to the degradation of bisretinoids and the release of toxic dicarbonyls (31, 41, 42). Moreover, as demonstrated in this study, the impairment of photoreceptor cells led to an acute increase in A2PE formation.

In this study we have investigated models of overload and iron chelation, and we report that both lead to measurable increases in one or more bisretinoids. At first, these results may appear incongruous. In one case, iron chelation increased bisretinoid levels (26) (**Figure 3**) due to diminished iron-catalyzed cleavage and the loss of bisretinoids, similar to that of antioxidants inhibiting bisretinoid consumption, which was due to photooxidation (31, 43–45). However, we questioned why an early increase in the fluorescent diretinal A2PE levels was observed in the model of retinal iron overload we employed. A2PE is the precursor of A2E and is a signature of photoreceptor cell outer segment bisretinoid; the levels of other bisretinoids found in RPE (A2E, A2-GPE) were not increased. Concurrent photoreceptor cell degeneration was evidenced by a reduction in ONL thickness in the histologic sections and by hyperreflectivity in the photoreceptor cell-attributable bands of SD-OCT scans. Hyperfluorescent spots were also visible in the fundus images. We therefore suggest that photoreceptor cell impairment was associated with the increase in A2PE levels.

In *ABCA4*-related disease, accelerated toxic bisretinoid formation in photoreceptor outer segments is a direct effect of ABCA4 protein insufficiency (46). The phenotype of retinol dehydrogenase 12 deficiency is similarly a primary effect of the gene defect (47). However, we have observed other conditions under which increased bisretinoid formation occurs as a secondary feature of photoreceptor cell dysfunctioning. For instance, qAF is increased at the advancing front of photoreceptor cell degeneration in acute zonal occult outer retinopathy and in central serous chorioretinopathy (48, 49). Abnormally increased SW-AF is also associated with outer segments that form the core of photoreceptor cell rosettes in degenerating mouse retina (50). In testing the premise that aberrant SW-AF can be a sign that photoreceptor cells are incapacitated, we observed a 36% increase in the SW-AF intensity 3 days after injecting NaIO_3_ into the mice; this increase was also related to ONL thinning, which is indicative of declining photoreceptor cell function and viability (51). By way of explaining the proposed link between photoreceptor cell impairment and increased bisretinoid formation, we suggest that perhaps the cell cannot meet the demand for the NADPH needed to reduce retinaldehyde to retinol to limit bisretinoid formation. Indeed, there may be competition for NADPH. NADPH is also the major reducing equivalent needed to return oxidized glutathione disulfide (GSSG) to reduced glutathione (GSH), enabling the continued availability of GSH for the cessation of lipid peroxidation. Under the conditions of iron accumulation, this activity is even more critical since redox-active iron in the cytosol accelerates free radical formation via the Fenton reaction, and the *OH radical then abstracts a hydrogen atom to initiate lipid peroxidation.

DFP is a water-soluble iron chelator that preferentially binds ferric ions (Fe^3+^) in a 3 : 1 ratio (DFP : iron). We previously reported that DFP-treated and non-treated mice exhibited similar postbleach recovery of the electroretinographic b-wave amplitude after photobleaching (90%). The treated mice were treated by ingesting DFP in their drinking water for 1 month as young adults. In this study we provide additional evidence that DFP, even when delivered to mice from birth until they were 2 months old, did not impair the functioning of *Rpe*65. Specifically, DFP-mediated iron chelation did not affect the regeneration of 11-*cis*-retinal 2 h after a 90% bleach in BALB/cJ mice.

As discussed above, the treatment of wild-type (C57BL/6 J) and *Abca4* ^–/–^ mice using DFP conferred an increase in bisretinoid levels due to diminished iron-catalyzed cleavage of bisretinoids (26) (**Figure 3**). Conversely, in DFP-treated *Mertk* ^–/–^ mice, the bisretinoid levels were decreased rather than increased. We note that in *Mertk* ^–/–^ mice, the photoreceptor cells degenerated due to RPE phagocytosis failure and the bisretinoids accumulated in the subretinal space rather than intracellularly. DFP can readily diffuse through the membrane, and it is expected that it would have subretinal access. It is significant that iron levels in the RPE and neural retina were elevated in the retinal dystrophic RCS rat (6) that carried a mutation in *Mertk* (52). This could be because owing to photoreceptor cell dysfunctioning and degeneration, iron homeostasis was impaired such that DFP did not chelate sufficiently. By analogy, iron was found to be increased in the eyes of AMD patients and those with *ABCA4*-related disease (25, 53). Alternatively, or in addition, DFP may carry iron into the subretinal debris where the iron may be released and generate the products of the Fenton reaction.

In summary, we found that compromised photoreceptor cell function due to increased iron was evidenced by ONL thinning and the hyperreflectivity of photoreceptor-attributable bands in the SD-OCT images and resulted in increased levels of bisretinoid A2PE and the enhanced appearance of hyperautofluorescent puncta in fundus AF images. These aberrations were more pronounced when iron was delivered to *Abca4^–/–^* mice. We also conclude that DFP chelation in mice did not interfere with *Rpe*65 activity.

## Data availability statement

The original contributions presented in the study are included in the article/Supplementary Material. Further inquiries can be directed to the corresponding author.

## Ethics statement

The animal study was approved by Animal Care and Use Committees at Columbia University and University of Pennsylvania. The study was conducted in accordance with the local legislation and institutional requirements.

## Author contributions

JZ: Data curation, Formal Analysis, Investigation, Methodology, Validation, Writing – review & editing. HK: Data curation, Formal Analysis, Investigation, Methodology, Validation, Writing – review & editing. DM: Data curation, Formal Analysis, Investigation, Methodology, Validation, Writing – review & editing. JD: Conceptualization, Writing – review & editing. JS: Conceptualization, Funding acquisition, Project administration, Supervision, Validation, Writing – original draft, Writing – review & editing.
